# Cisplatin-associated ototoxicity amongst cervical cancer patients: A prospective cohort study in south Africa

**DOI:** 10.1371/journal.pone.0283639

**Published:** 2023-04-04

**Authors:** Jessica Paken, Cyril Devadas Govender, Mershen Pillay, Merga Feyasa, Vikash Sewram

**Affiliations:** 1 Discipline of Audiology, School of Health Sciences, University of KwaZulu-Natal, Durban, South Africa; 2 Division of Epidemiology & Biostatistics, Department of Global Health, Faculty of Medicine and Health Sciences, Stellenbosch University, Cape Town, South Africa; 3 Department of Global Health, Faculty of Medicine and Health Sciences, African Cancer Institute, Stellenbosch University, Cape Town, South Africa; University of Catania, ITALY

## Abstract

**Background:**

Concurrent chemoradiotherapy using weekly cisplatin remains standard of care for locally advanced cervical cancer in Sub-Saharan Africa. While cisplatin remains a popular cancer chemotherapeutic, it has an irreversible ototoxic effect on patients’ auditory system. However, there is a paucity of epidemiological information on its extent and severity during cervical cancer treatment. In a region with a high burden of cervical cancer, this has serious consequences for aural intervention and rehabilitation.

**Methods and findings:**

Using a prospective cohort study design, 82 patients with incident cervical cancer, receiving weekly cisplatin chemotherapy (50 mg/m^2^ body surface) at a tertiary level hospital in KwaZulu-Natal Province of South Africa, underwent audiological assessments at various intervals. We describe the temporal impact of cisplatin exposure on hearing loss, its combined effect with HIV-infection, and estimate ototoxicity incidence in this cohort. The median age was 52 years with Stages IIB (45%) and IIIB (35.4%) cancers being most common. Complaints of reduced hearing sensitivity increased significantly (p<0.0001). Bilateral, asymmetrical sensorineural hearing loss, with greater effect in the extended high-frequency range, was evident. Cisplatin dosage was significantly associated with ototoxicity severity at one- (p = 0.017), three- (p = 0.010), and six-month (p = 0.015) post-treatment follow-up. HIV-seropositivity (53.7%) was significantly associated with NCI-CTCAE Grading Scale at three- (p = 0.022) and six-months (p = 0.023) post-treatment. Multiple Tobit regression revealed a cumulative dose effect bilaterally, after adjustment for age and HIV status, evident from 9000Hz and above in the right ear, while a plateau effect was observed at 250mg/m^2^ in the left ear. The incidence was ototoxicity was 98% at a cumulative dose of 150mg/m^2^.

**Conclusion:**

The findings of this epidemiologic study highlight the temporal course and severity of ototoxicity experienced by cervical cancer patients treated with cisplatin, with greater impact in HIV-positive subgroup, thus underscores the need for audiological monitoring and timely interventions in this cohort.

## Introduction

Cervical cancer continues to significantly burden the Sub-Saharan continent with the highest regional incidence [1]. Based on 2020 estimates, a 49.5% increase in incidence by 2040 is projected [2]. In South Africa, it is the second most common cancer among females with an age-standardised incident rate of 23.71 per 100 000 [3]. It is, however, the most common cancer amongst the black female population, with an age-standardized incidence rate of 28.25 per 100 000 and a lifetime risk of 1 in 33. Locally advanced cervical cancer (LACC) incidence is also associated with HIV positivity resulting in severe morbidity [4]. Modalities of treatment can include surgery, radiotherapy, chemotherapy, or a combination thereof, based on the International Federation of Gynecology and Obstetrics (FIGO) stage [5]. Concurrent chemoradiotherapy using weekly cisplatin as the chemotherapeutic agent is the standard of care for locally advanced cervical cancer (LACC), commonly diagnosed in our setting [6].

While cisplatin is one of the most popular anti-cancer drugs due to its effectiveness, it is known to have a diverse toxicity profile affecting the gastro-intestinal, renal [7], hematologic [8], and auditory system [9]. Although cisplatin’s side-effect on other systems can be minimized or even reversed, its impact on the auditory system, due to the functional and cellular damage of the inner ear [10, 11], is medically untreatable or unpreventable [12], and is referred to as ototoxicity.

Cisplatin-associated ototoxicity is characterized by a pattern of bilateral, progressive sensorineural hearing loss, with a greater influence on the high frequencies [13–16]. The incidence of cisplatin-associated ototoxicity is highly variable (21% - 80%) [17] due to several factors related to (a) patient characteristics (age, co-morbidities, pre-existing hearing loss, patient physiology), (b) environment (noise exposure) and (c) cisplatin exposure parameters (dosage, administration and treatment duration) [18].

Hearing loss is also often a common occurrence of diabetes and hypertension, due to either the disease pathophysiology or the medication used in the treatment of these conditions. Diabetes-related-auditory manifestations are due to “spiral ganglion atrophy, degeneration of the vestibulocochlear myelin sheath, reduction of the number of spiral lamina nerve fibres, the thickening of the capillary walls of the stria vascularis and small arteries” [19]. Hypertension-related hearing loss may, on the other hand, results from inner ear haemorrhage, tissue hypoxia, or ionic changes in cell potentials; however, it also results in high-frequency sensorineural hearing loss [20]. Hearing loss due to the disease pathophysiology of diabetes or hypertension can be further worsened by the use of certain antiretroviral treatments (ARTs), as a result of mitochondrial toxicity, which may result in accelerated changes in the cochlear and/or central auditory system [21].

Given the high incidence of cervical cancer in Sub-Saharan Africa, the associated comorbidities described above and the resulting poor quality of life in affected patients that may be further exacerbated by hearing loss, this prospective cohort study aimed to evaluate the extent and severity of cisplatin-associated ototoxicity experienced by cervical cancer patients in South Africa.

## Methodology

### Participants and chemotherapy treatment

Ethical clearance was obtained from the University of KwaZulu-Natal Biomedical and Research Ethics Committee (BE064/13) and the participating hospital, a tertiary level hospital, and one of the main referral centers for cancer patients housing an audiology department in KwaZulu-Natal (KZN), South Africa. Following written informed consent, 82 women diagnosed with histologically confirmed incident cervical cancer were prospectively enrolled into the study before treatment, within two years. Women presenting with profound hearing loss at baseline assessment, previous exposure to cisplatin chemotherapy, or with a history of tuberculosis, and/or malaria were excluded. Of the 82 participants, 80 attended all follow-up appointments. Attrition in the study was due to participant death. Chemotherapy consisted of cisplatin (50 mg/m^2^ body surface weekly for six cycles) in combination with dexamethasone (48 mg) and ondansetron (24 mg) offered as outpatient therapy. In some cases, the number of chemotherapy cycles was reduced due to some patients experiencing side effects i.e. severe gastrointestinal disturbances such as nausea, vomiting, and/or diarrhoea. Patients with cervix carcinoma were routinely tested for HIV during diagnostic work-up. All patients identified with reduced hearing ability were counseled regarding aural rehabilitation and referred for further interventions.

### Data collection

Participants’ medical records were reviewed to gather demographic and clinico-pathologic information including medical history. Cervical cancer was assessed using the International Federation of Gynecology and Obstetrics (FIGO) staging system. All participants underwent audiological assessments before commencing cisplatin chemotherapy, with follow-up assessments at the beginning of the fourth cycle, and at one-, three-, and six-month post-treatment. These assessments included a case history, otoscopic examination, immittance audiometry (i.e. tympanometry, and ipsilateral and contralateral acoustic reflex threshold testing), pure tone audiometry (including masking where required), extended high-frequency audiometry (up to 20000Hz), speech reception threshold (SRT) testing, word recognition score (WRS) testing and distortion product otoacoustic emission (DPOAE) testing, as prescribed for ototoxicity monitoring by the American Speech-Language and Hearing Association (ASHA) [22]. The case history interview focused on the participant’s hearing, medical, and family history, and exposure to noisy environments. Questions on tinnitus were adapted from the Tinnitus Ototoxicity Monitoring Interview (TOMI) schedule [23]. An Agine otoscope was used to conduct otoscopic examinations, while the GSI Tympstar V2 Impedance meter was used for immittance audiometry. Pure tone and speech audiometry were conducted in a soundproof booth, using the Madsen Astera. The Maico Otoacoustic emissions were used for the acquisition of DPOAE data. Stimulus parameters for the DPOAE include a 6-frequency range of 1500, 2000, 3000, 4000, 5000 and 6000Hz, with L1/L2 intensity of 65/55dB at a sampling rate of 31.250Hz. A detailed description of the audiological assessment protocol, research tools and normative data, is available in Paken, Govender [24, 25].

### Data analysis

Patient characteristics were summarized using frequencies and proportions for categorical and/or binary variables, whilst medians and interquartile ranges for asymmetric distributions. The distribution of participant’s pure-tone air conduction thresholds at the different frequencies was analysed separately for left and right ears, to avoid mixing frequencies, which have different upper cut-off limits on the audiometer. Data analysis is per ear and not per patient to prevent the effects of correlation between the ears, as ototoxic hearing loss is reported to be bilateral. For example, a higher pure tone threshold in the right ear may affect the threshold in the left ear and vice versa.

Classification of hearing loss was based on frequencies in the range 125Hz– 8000Hz, as high-frequency audiometry does not have standardized results [26]. The extended high-frequency range (9000Hz– 20000Hz) was used for comparative purposes, with each patient serving as their control. The degree of hearing loss was classified based on the audiometric thresholds, namely, mild (26-40dB), moderate (41-55dB), moderately severe (56 -70dB), severe (71-90dB) and profound (>90dB) [27]. The type of hearing loss was further categorized into conductive, sensorineural, and mixed, based on a comparison of the air and bone conduction thresholds, with a) air and bone conduction thresholds within normal limits indicating normal peripheral hearing, b) air and bone conduction depressed in the absence of an air-bone-gap indicating sensorineural hearing loss, and c) air and bone conduction depressed in the presence of an air-bone-gap indicating mixed hearing loss [28].

The Cochran-Armitage test for trend was used to test for trend in binomial proportions of auditory complaints over time. ASHA criteria [22] was used for determining the presence of an ototoxic shift, with an extension to the extended high-frequency range as recommended by the AAA [29] whilst severity of ototoxicity in affected patients was graded using the National Cancer Institute (NCI) Common Terminology Criteria for Adverse Events (CTCAE) version 5 [30] (S1 File). According to ASHA [22], the following would represent a significant hearing loss:

- ≥ 20dB decrease at any one test frequency,
- ≥10dB decrease at any two adjacent frequencies, or
- loss of response at three consecutive frequencies where responses were previously obtained. Repeated Measures Analysis of Variance (ANOVA) was used to determine if the difference in mean DPOAE amplitudes between 1500Hz - 6000Hz was statistically significant at each audiological evaluation.

The Fisher’s exact test was used to determine the association between cisplatin dosage (Low; 150 and 200mg/m^2^ versus High, 250mg/m^2^ and 300mg/m^2^) and HIV seropositivity on the severity of ototoxicity. As a result of the right censoring in pure-tone frequency threshold measurements (outcome variable), due to the upper limits of the audiometer at the various frequencies (125Hz-14000Hz), Tobit regression was employed to examine the association between the outcome variable and cumulative cisplatin dosage (mg/m^2^) (predictor variable). This approach was key as the number of ‘No Responses’ in the extended high-frequency range increased at each successive audiological evaluation (S2 File). Furthermore, the frequencies of 16000Hz, 18000Hz, and 20000Hz were not included in the Tobit regression model due to the extremely large number of ‘No Responses’. The models for each ear were adjusted for the effects of age and HIV seropositivity. Stratification by HIV-status further allowed for the evaluation of a dose-effect between HIV-positive and HIV-negative participants. The model coefficients indicated how changes in the dosage of cisplatin related to changes in pure tone frequency threshold measurements. With respect to sample size calculation, 78 participants were found adequate to achieve 80% power to detect a moderate effect size (W) of 0.3 using 2 degrees of freedom (i.e. 2 groups) with a significance level (α) of 0.05. However, 82 participants were targeted to compensate for attrition during the study. G*Power Version 3.1.9.4 (Universität Kiel, Germany) was used to estimate sample size for linear regression. Parameters for a medium effect size (F^2^) of 0.15, α = 0.05, power = 80% and 3 predictors (i.e. age, HIV-seropositivity, and cisplatin cumulative dosage) resulted in a sample size of 77. All statistical tests were 2-sided with statistical significance defined as p-value ≤ 0.05. All analyses were conducted in Stata version 15 (StataCorp Inc., College Station, TX, USA).

## Results

### Participant characteristics

Participant demographic and medical characteristics are presented in Table 1. The median age of the cohort was 52 years (IQR = 43–60). Stage IIB (45%) and Stage IIIB (35.4%) cancers were the most common. The five patients with stage 1 cancer received chemotherapy due to peri-neural involvement on histology, lymph node involvement post-surgery, whilst another developed a local recurrence post-hysterectomy. Seventy-five participants (91.5%) were of Black African descent. A larger proportion of the participants were HIV positive (53.7%) and upon further evaluation of co-morbid conditions, exclusive HIV infection was present amongst 45.1% of the participants whilst 18.3% was reported as having hypertension exclusively. Diabetes was the least common (12.2%), either exclusively or in combination. Only thirty-two participants (39.1%) completed all 6 cycles of chemotherapy (cisplatin cumulative dose of 300 mg/m^2^) while the remaining participants all received either 150 mg/m^2^, 200 mg/m^2^ or 250 mg/m^2^ of cisplatin, respectively. At each case history ascertainment, all participants reported no ongoing exposure to high levels of recreational and occupational noise. Furthermore, a review of the medical file and patient reports revealed normal renal function in all participants.

**Table 1:** Demographic and medical characteristics of the participants, n (%).

Patient characteristics	Number, n (%)
**Age**	
< = 40	12 (14.6)
41–50	24 (29.3)
51–60	26 (31.7)
>60	20 (24.2)
**Ethnic group**	
Black African	75 (91.5)
Indian/Asian	4 (4.9)
Coloured	3 (3.6)
**Cancer Stage**	
I A	3 (3.7)
I B	2 (2.4)
II A	9 (10.9)
II B	37 (45.1)
III A	2 (2.4)
III B	29 (35.4)
**HIV Status**	
Positive	44 (53.7)
Negative	38 (46.3)
**Co-morbidities**	
HIV only	37 (45.1)
Hypertension only	15 (18.3)
Diabetes and Hypertension	6 (7.3)
HIV and Hypertension	4 (4.9)
Diabetes only	2 (2.4)
HIV, Diabetes and Hypertension	2 (2.4)
HIV and Epilepsy	1 (1.2)
Hypertension and Arthritis	1 (1.2)
None	14 (17.1)
**Cumulative cisplatin dosage (mg/m** ^**2**^ **)**	
150 (3 cycles)	17 (20.7)
200 (4 cycles)	17 (20.7)
250 (5 cycles)	16 (19.5)
300 (6 cycles)	32 (39.1)

### Self-reported audiological symptoms

A summary of the self-reported audiological symptoms is presented in Table 2. There was a significant increase (p≤0.001) in the number of participants reporting reduced hearing sensitivity at each successive audiological evaluation, with bilateral hearing difficulties being more frequently reported than unilateral. Whilst complaints of otalgia and aural fullness increased during cisplatin treatment, but steadily decreased post-cisplatin-treatment, these changes were found not to be statistically significant. Although, a similar pattern was evident with complaints of tinnitus, this change was however statistically significant across the follow-up period (p≤0.001), with high-pitched tinnitus, the most common at all audiological evaluations, persisting in seven of the 28 participants (25%) who reported this symptom at baseline.

**Table 2:** Self-reported audiological symptoms.

Self-reported Audiological symptoms	Follow-up, n (%)					Test for Trend (p-value)
	Baseline	Mid-cycle	1-month post-treatment	3-month post-treatment	6-month post-treatment	
**Experience of hearing difficulties**	9 (11.0)	13 (15.9)	18 (21.9)	18 (21.9)	26 (31.7)	0.001*
**Ear in which difficulties are experienced**						
Both	5 (55.6)	8 (61.5)	10 (58.8)	12 (66.6)	15 (60.0)	
Left	3 (33.3)	4 (30.8)	3 (17.7)	3 (16.7)	5 (20.0)	
Right	1 (11.1)	1 (7.7)	4 (23.5)	3 (16.7)	5 (20.0)	
**Complaints of otalgia**	5 (6.1)	9 (11.0)	10 (12.2)	4 (4.9)	3 (3.7)	0.260
**Complaints of aural fullness**	4 (4.9)	10 (12.2)	9 (11.0)	3 (3.85)	3 (3.7)	0.240
**Complaints of tinnitus**	28 (34.1)	36 (43.9)	24 (29.3)	18 (21.2)	10 (12.2)	0.001*
**Description of tinnitus**						
High-pitched	25 (89.3)	28 (77.7)	17 (70.8)	14 (77.8)	7 (70.0)	
Low-pitched	2 (7.1)	2 (5.6)	2 (8.3)	1 (5.6)	0 (0)	
Pulsating	1 (3.6)	5 (13.9)	5 (20.8)	3 (16.7)	1 (10.0)	
Roaring	0 (0)	1 (2.8)	0 (0)	0 (0)	2 (20.0)	
**History of repeated ear infections**	2 (2.4)	2 (2.4)	2 (2.4)	2 (2.4)	2 (2.4)	

*Cochran-Armitage Test for Trend indicates a significant trend in complaints associated with hearing difficulties and tinnitus over time.

### Description of clinical hearing loss

Abnormal otoscopic findings (in this case tympanic membrane perforation) were evident in the right ear of one participant, and the left ear of another participant at all audiological evaluations. At all audiological evaluations, 99% of the participants presented with Type A tympanograms bilaterally based on Jerger (1970) [31] classification for the normal limits for middle ear pressure, ear canal volume, and static compliance. These tympanograms together with the acoustic reflex thresholds within normal limits were suggestive of normal middle ear function. There was a significant increase in the number of participants presenting with bilateral hearing loss from baseline at each successive audiological evaluation (p = 0.04) (Table 3). This increased from approximately 20.1% at baseline to 38.8% at the six-month post-treatment evaluation (p = 0.04). Sensorineural hearing loss was most common at all audiological evaluations bilaterally, with an increase at each follow-up. Mild hearing loss was the most common in the left ear while mild-to-moderate hearing loss was the most common in the right ear at each assessment.

**Table 3:** Characteristics of hearing loss as indicated by pure tone thresholds.

	Baseline	Mid-cycle	One-month post-treatment	Three-month post-treatment	Six-month post-treatment	p-value
**Number of participants presenting with hearing loss, n (%)**						
Both ears	17(20.7)	21 (25.6)	24 (29.2)	24 (29.2)	31 (37.8)	0.040*
Left ear only	6 (7.3)	7 (8.5)	4 (4.9)	10 (12.2)	10 (12.2)	0.550
Right ear only	5 (6.1)	6 (7.3)	6 (7.3)	5 (6.1)	3 (3.7)	0.130
**Types of Hearing loss, n (%)**						
Sensorineural						
Right ear	21 (25.6)	25 (30.5)	28 (34.1)	28 (34.2)	33 (40.2)	
Left ear	22 (26.8)	27 (32.9)	27 (32.9)	34 (41.5)	39 (47.6)	
Mixed						
Right ear	1 (1.2)	2 (2.4)	1 (1.2)	1 (1.2)	1 (1.2)	
Left ear	1 (1.2)	1 (1.2)	0 (0)	0 (0)	0 (0)	
**Degree of Hearing Loss, n (%)**						
Right ear						
Normal	60 (73.2)	55 (67.1)	51 (62.2)	51 (63.8)	46 (56.1)	
Mild	8 (9.8)	8 (9.8)	11 (13.4)	8 (9.8)	11 (13.4)	
Mild-to-moderate	7 (8.5)	9 (11.0)	9 (11.0)	12 (14.6)	13 (15.9)	
Mild-to- moderately severe	5 (6.1)	7 (8.5)	5 (6.1)	6 (7.3)	6 (7.3)	
Mild-to-severe	1 (1.2)	0 (0)	1 (1.2)	1 (1.3)	1 (1.3)	
Mild-to-profound	1 (1.2)	0 (0)	1 (1.2)	1 (1.3)	1 (1.3)	
Moderate-to-moderately severe	0 (0)	0 (0)	0 (0)	1 (1.3)	1 (1.3)	
Moderate-to- profound	0 (0)	1 (1.2)	1 (1.2)	1 (1.3)	1 (1.3)	
Left ear						
Normal	59 (72)	54 (65.9)	53 (64.6)	46 (57.5)	41 (51.3)	
Mild	8 (9.8)	11 (13.4)	10 (12.2)	17 (20.7)	16 (19.5)	
Mild-to-moderate	6 (7.3)	8 (9.8)	9 (11.0)	7 (8.6)	8 (9.8)	
Mild-to-moderately severe	3 (3.7)	4 (4.9)	4 (4.9)	6 (7.3)	9 (11.0)	
Mild-to-severe	3 (3.7)	3 (3.7)	3 (3.7)	3 (3.7)	3 (3.7)	
Moderate	1 (1.2)	0 (0)	0 (0)	0 (0)	0 (0)	
Moderate-to-moderately severe	0 (0)	0 (0)	0 (0)	0 (0)	2 (2.4)	
Severe-to-profound	2 (2.4)	2 (2.4)	1 (1.2)	1 (1.2)	1 (1.2)	

*Cochran-Armitage Test for Trend revealed a significant increase in the number of participants presenting with clinical and ototoxic hearing loss bilaterally over time.

**Fig 1:**
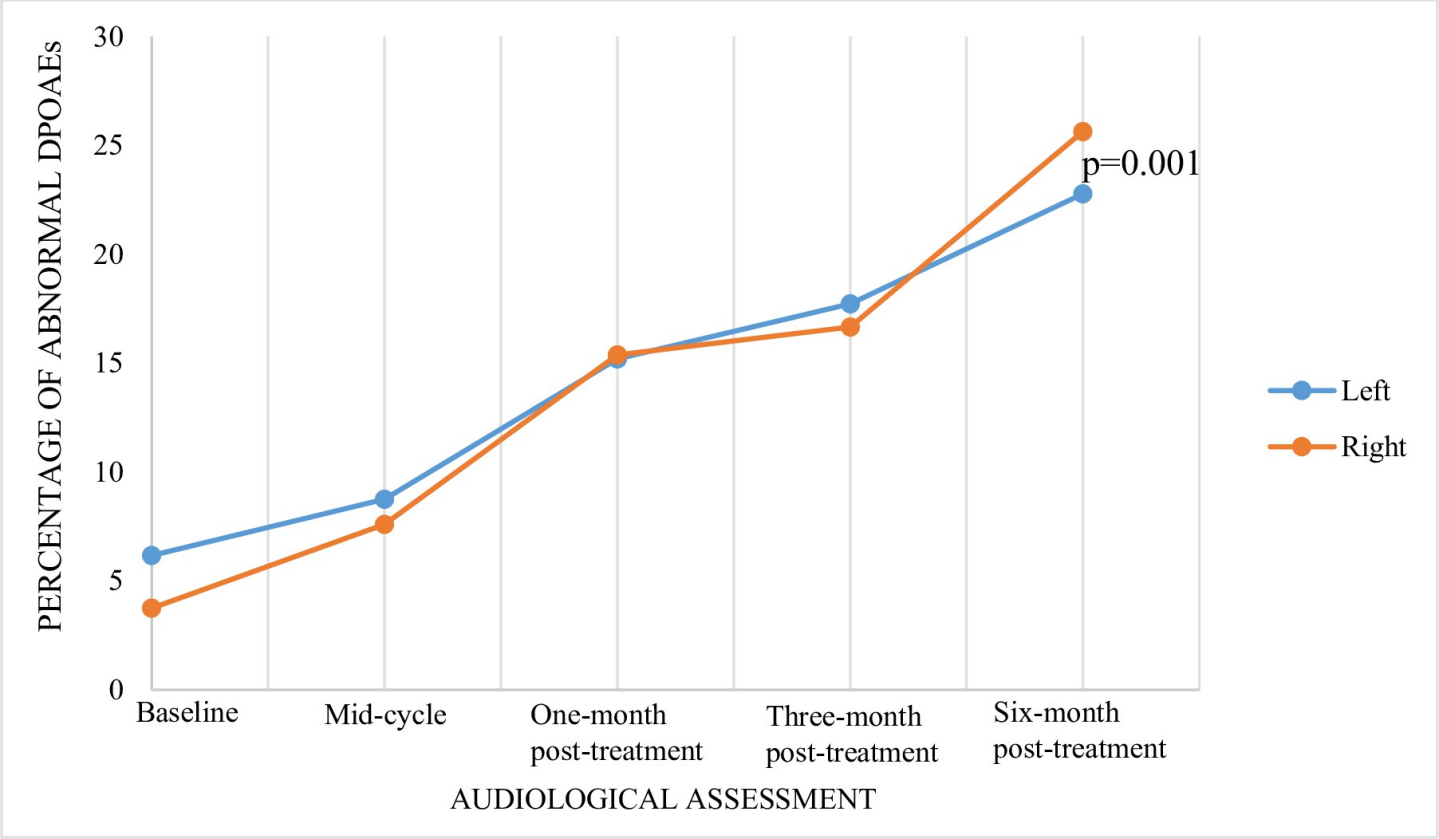
Percentage of abnormal Distort Product Oto-acoustic Emissions (DPOAEs) at each audiological evaluation. There is a monotonic increase (p = 0.001) in the number of abnormal DPOAEs at each audiological visit.

There was a significant increase in the number of individuals presenting with abnormal DPOAEs bilaterally at each successive audiological evaluation (p = 0.001 bilaterally) (Fig 1). DPOAEs were considered present if there was an amplitude of ≥ 0dBSPL and/or a difference of more than 5dBSPL between the DPOAE and noise floor [Signal-to-noise-ratio] [32].

**Fig 2:**
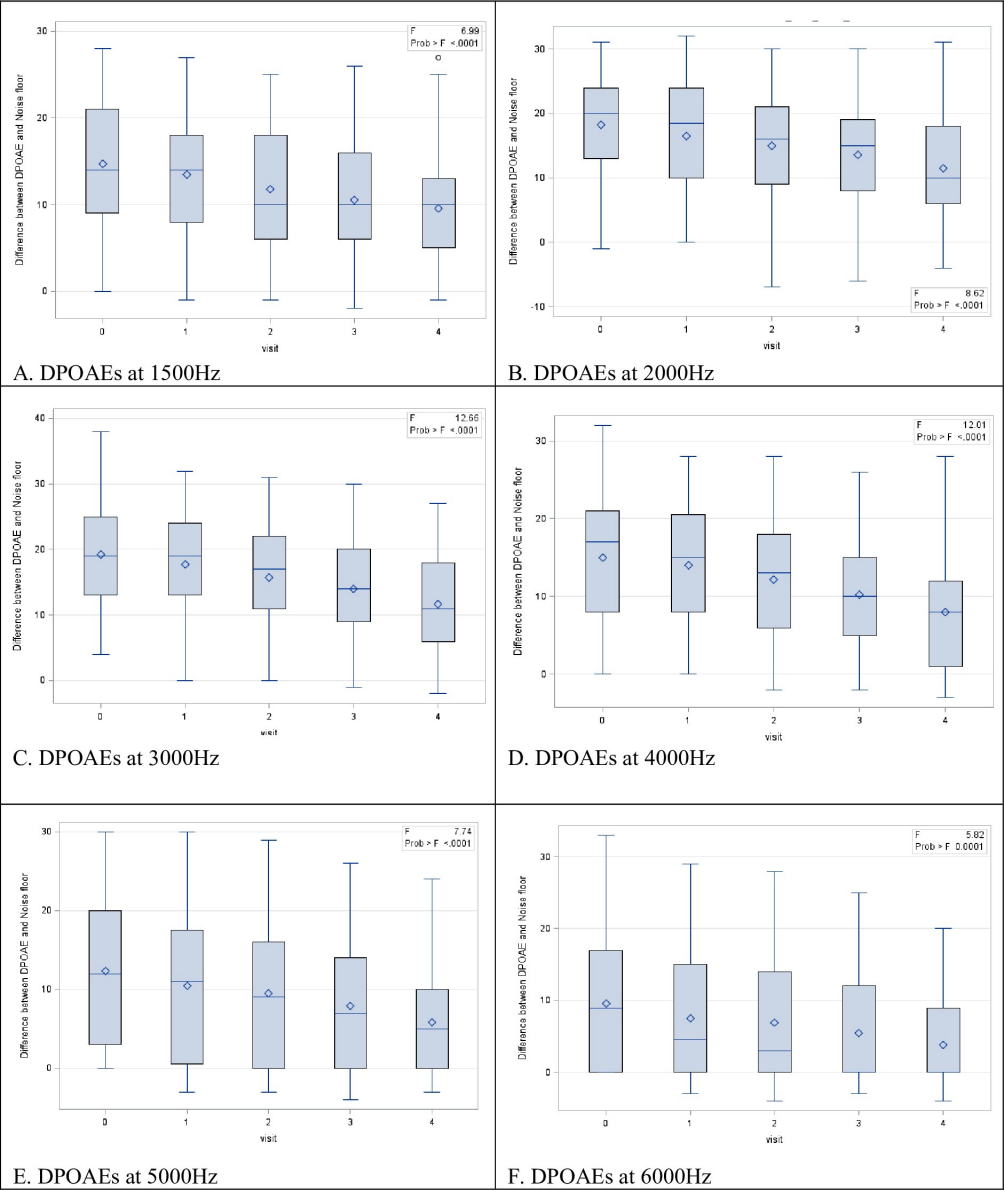
Box plots reflecting the change in the difference between the DPOAE and the noise floor at all frequencies in the right ear. ANOVA test revealed a significant decrease (p<0.05) in the difference between the DPOAE and noise floor at each audiological evaluation.

**Fig 3:**
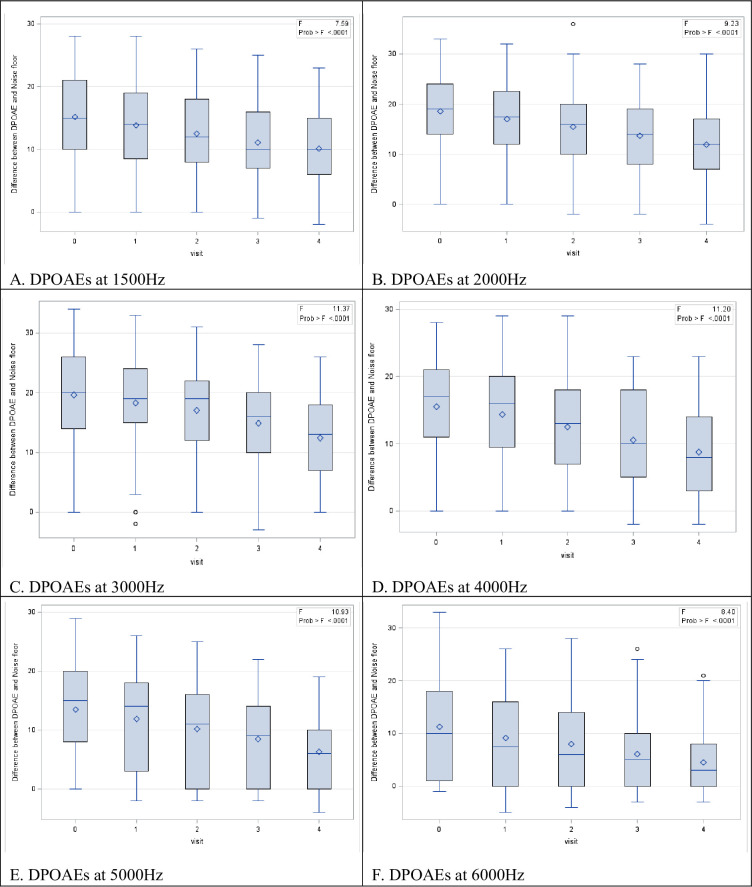
Box plots reflecting the change in the difference between the DPOAE and the noise floor at all frequencies in the left ear. Footnote: ANOVA test revealed a significant decrease (p<0.05) in the difference between the noise floor and DPOAE at subsequent audiological visits.

There was a consistent decrease in the DPOAE amplitude bilaterally at each follow-up, as evidenced by the median DPOAE profiles with the decrease being more pronounced at the higher frequencies for both the right (Fig 2) and left (Fig 3) ear.

Speech audiometry revealed that more than 79 participants (96%) presented with good SRT-PTA correlation bilaterally at each time point; therefore, validating the pure tone audiometry test results. Word recognition scores testing revealed that 75 participants (>95%) presented with excellent word recognition ability within normal conversational levels (below 60 dB HL) bilaterally at each audiological evaluation.

The ASHA criteria were used to determine the presence of an ototoxic shift in the patient’s hearing at each audiological evaluation. Given that any of the three criteria need to be fulfilled in the assessment for ototoxicity, Table 4 outlines the proportion of patients meeting each of the criteria during follow-up. The second criteria, i.e. ≥ 10 dB shift at 2 consecutive frequencies, was met by 98% of the patients. At the one-month post-treatment follow-up, whilst two patients passed away due to illness, the remaining two met the criteria for ototoxicity. At mid-cycle, 21 days after initiating treatment and exposure to a cumulative cisplatin dose of 150mg/m^2^, an incidence of ototoxicity of 98% was recorded in this cohort.

**Table 4:** Incidence of ototoxic shift according to ASHA criteria.

ASHA Criteria	Ototoxic Shift, n (%)			
	Mid-Cycle	One-month post-treatment	Three-month post-treatment	Six-month post-treatment
≥ 20 dB pure tone threshold shift at a single frequency	21 (26)	35 (43)	50 (61)	62 (76)
≥ 10 dB shift at 2 consecutive frequencies	80 (98)	80 (100)	80 (100)	80 (100)
threshold response shifting to “no response” at three consecutive frequencies	67 (82)	74 (90)	75 (91)	77 (94)
**Incidence of ototoxicity**	**98% after 21 days of commencing therapy (cumulative dose of 150 mg/m** ^**2**^ **)**			

**Fig 4:**
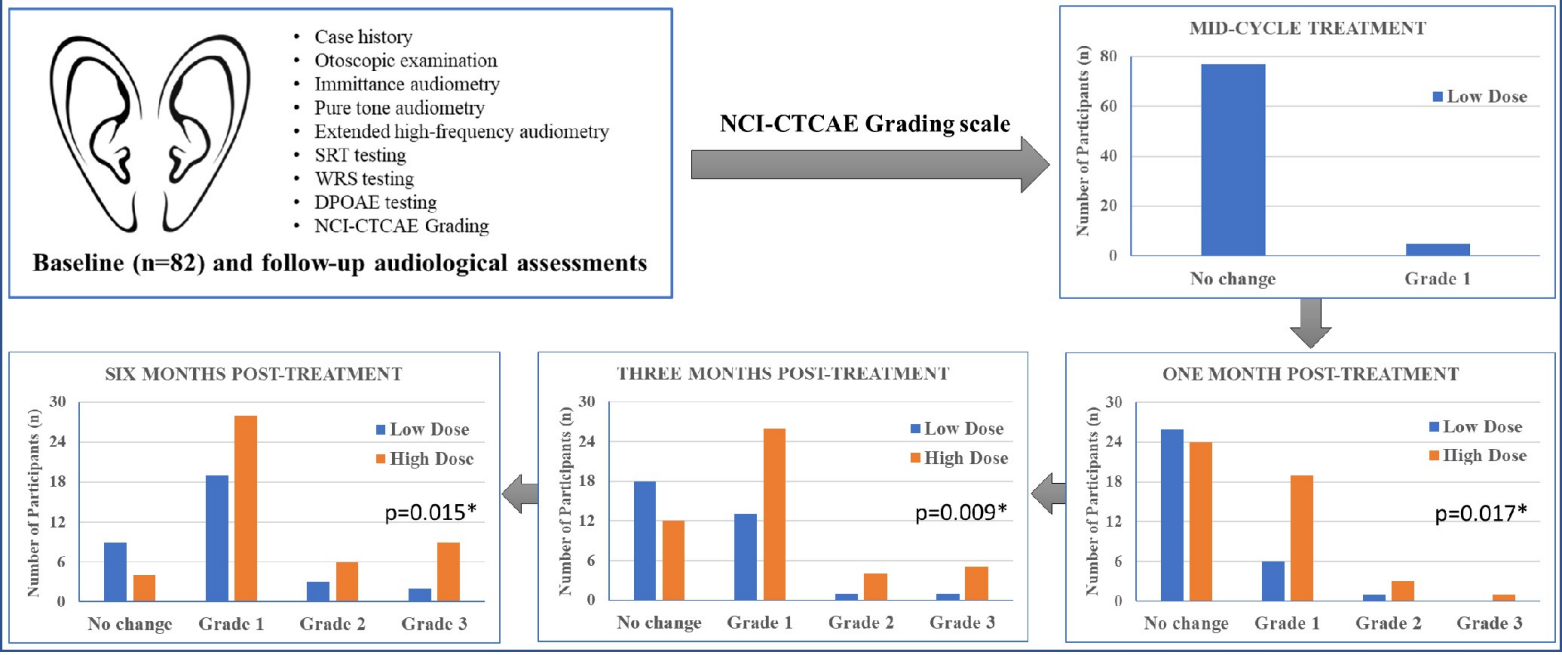
Temporal variation in severity of ototoxicity according to NCI-CTCAE classification. * denotes statistical significance in the relationship between Cisplatin dose and Grade (Fisher’s exact test) At mid-cycle, all patients received 3 cycles of cisplatin (150 mg/m^2^)–stratified as Low Dose only.

The deterioration in hearing thresholds during cisplatin-chemotherapy treatment and their ability to guide recommendations regarding hearing amplification was explored. The temporal association in the severity of ototoxicity (NCI-CTCAE grading scale) and cumulative cisplatin dosage, stratified into low (150 & 200 mg/m^2^) and high (250 & 300 mg/m^2^), respectively is illustrated in Fig 4. At mid-cycle 77 participants showed no change from the baseline assessment with 5 participants presenting with Grade 1 change despite all having received a cumulative dose of 150mg/m^2^. At subsequent follow-up assessments, it becomes evident that the severity of ototoxicity is associated with treatment doses as more participants in the higher dosage group begin to show greater ototoxic change. The temporal association between cisplatin dosage and ototoxicity severity is significant at one- (p = 0.017), three- (p = 0.009) and six-month (p = 0.015) post-cisplatin treatment. At the end of the 6-month follow-up, 15/47 (32%) of patients in the high-dose group were in the Grade 2/3 category compared to only 5/33 (15%) of the patients in the low-dose group.

The impact of HIV infection on the severity of ototoxicity using the NCI-CTCAE Grading Scale was also explored, and results are provided in Table 5. A significant increase in ototoxicity severity was observed amongst the HIV-positive participants at three (p = 0.022) and six months (p = 0.020) indicating that HIV-positive patients present with a greater degree of co-morbidity in addition to their cancer.

**Table 5:** Severity of ototoxicity by HIV status at each audiological assessment (NCI-CTCAE Grading Scale).

Follow-up	HIV status	NCI-CTCAE Grading Scale, n (%)				p-value
		No change	Grade 1	Grade 2	Grade 3	
Mid-cycle	Negative	35 (92.1)	3(7.9)	0	0	0.659
	Positive	42 (95.5)	2 (4.5)	0	0	
One-month post-treatment	Negative	26 (70.3)	10 (27.0)	0	1 (2.7)	0.124
	Positive	24 (55.8)	15 (34.9)	4 (9.3)	0	
Three-month post-treatment	Negative	20 (54.1)	13 (35.1)	1 (2.7)	3 (8.1)	0.022*
	Positive	10 (23.3)	26 (60.5)	4 (9.3)	3 (6.9)	
Six-month post-treatment	Negative	10 (27.0)	22 (59.4)	1 (2.7)	4 (10.8)	0.023*
	Positive	3 (7.0)	25 (58.1)	8 (18.6)	7 (16.3)	

*Fisher’s Exact Test revealed a significant association between HIV status and the NCI-CTCAE Grading Scale at three-and six-month post-cisplatin treatment.

**Fig 5:**
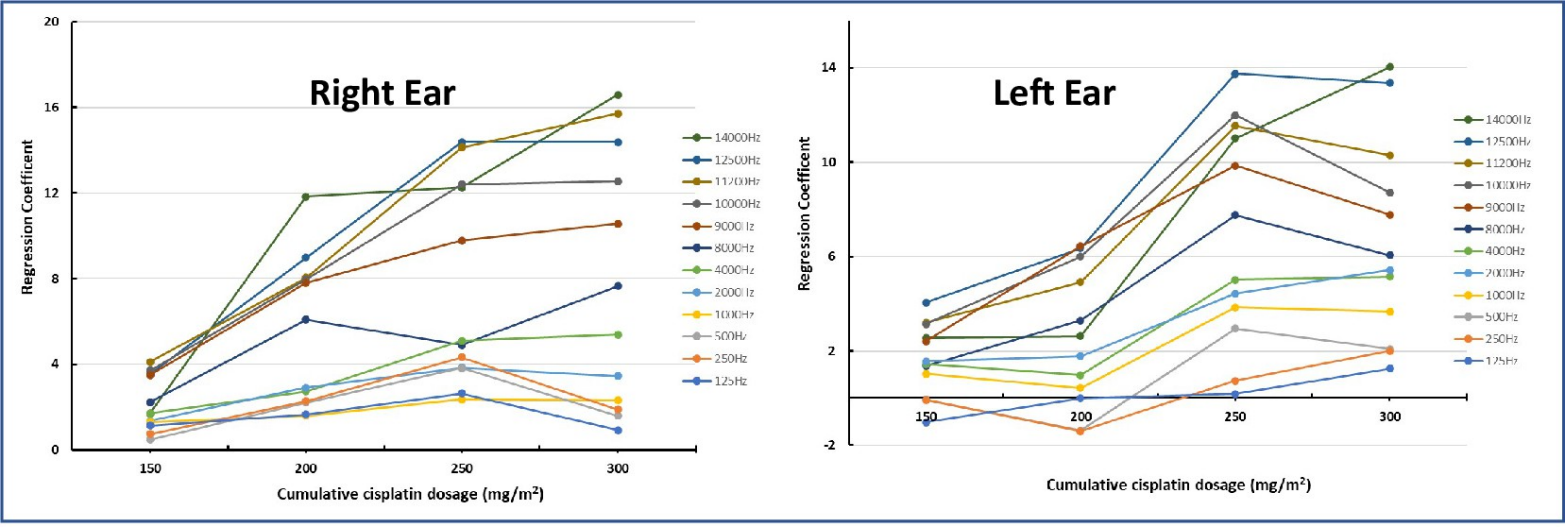
Influence of cumulative cisplatin dosage on hearing thresholds in the right and left ear, after adjustment for age and HIV status. Dose Effect is evident bilaterally. Clustering of extended high frequencies together and the conventional frequencies together is visible bilaterally. Plateau effect is visible at 250mg/m2 from 8000 to 14000Hz in the left ear.

Multiple Tobit regression was undertaken to evaluate the influence of cumulative cisplatin dosage on hearing thresholds at various frequencies (125Hz-14000Hz) in both the right and left ear, after adjustment for the effect of participant age and HIV status. Correlates of pure tone threshold measurements for the right ear revealed that significant changes were evident from 1000 Hz at a cumulative dose of 150 mg/m^2^, with the degree of change increasing across the various doses of cisplatin at each frequency. The β-coefficients provided in S3 File, indicate the magnitude of change for each of the covariates at the frequencies assessed. It is evident that higher frequencies are of greater significance in assessing hearing changes. A similar trend was observed with the left ear (S4 File), although the right ear was found to be more sensitive to ototoxic shifts. A significant trend (p≤ 0.05) between hearing shift and cisplatin dosage, especially at frequencies 9000Hz and above was observed for the right ear and this dose-response is evident in Fig 5. A clustering of the extended-high frequencies (9000Hz– 14 000Hz) is evident, with a possible plateau effect at 300mg/m^2^. A dose-response is also observed at higher frequencies with the left ear, but a notable decrease in the rate of hearing change becomes evident after a 250 mg/m^2^ dose.

**Fig 6:**
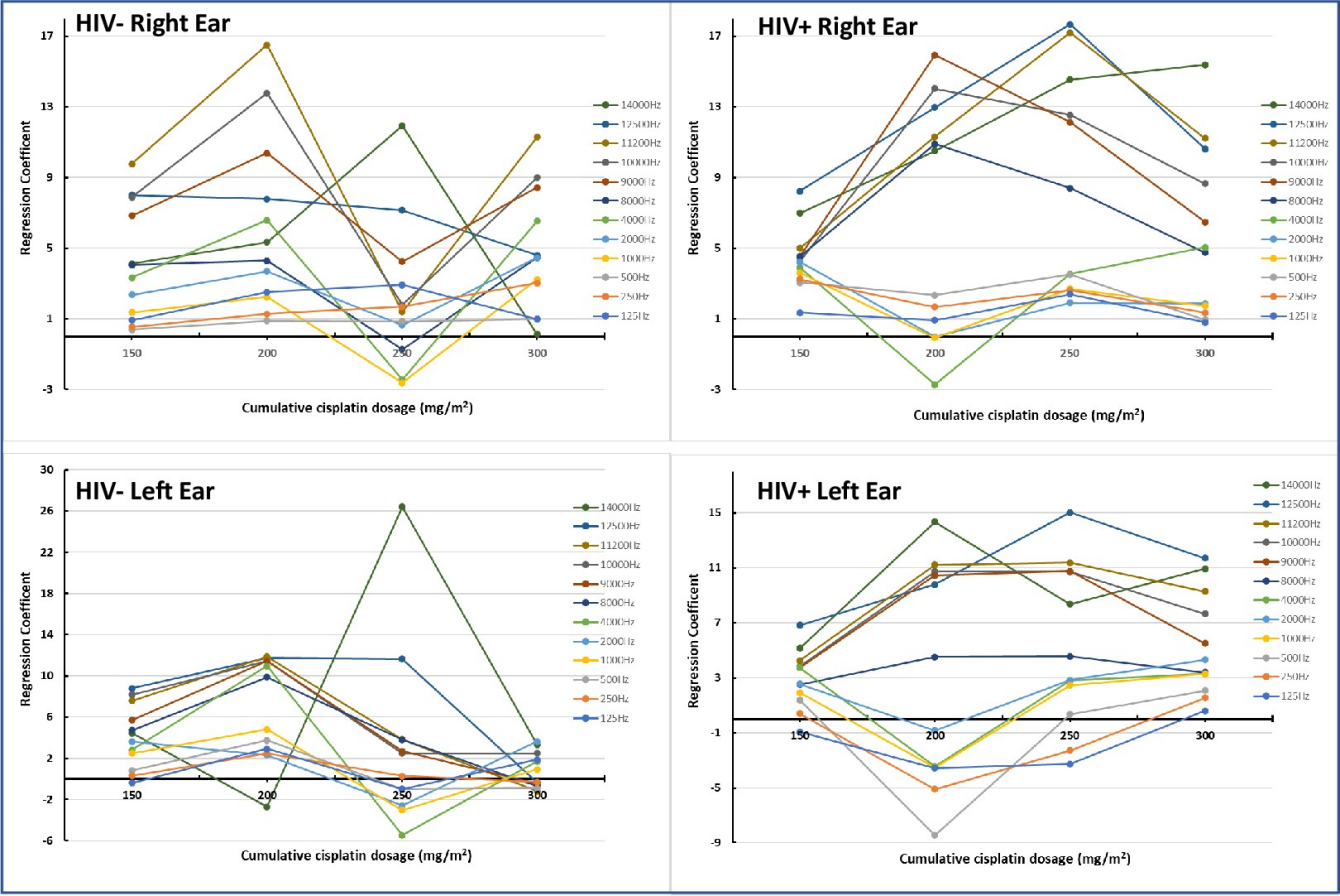
Influence of cumulative cisplatin dosage on hearing thresholds in the right and left ear in patients stratified by HIV status, after adjustment for age. Dose effect evident in HIV positive group only. The change in hearing threshold is also reached more quickly with a lower dosage in the HIV positive group.

The impact of cumulative cisplatin dosage on hearing thresholds was also evaluated separately between HIV-positive and HIV-negative participants, whilst controlling for the effect of age. The β-coefficients for this model are provided in S5–S8 Files. It was also evident that for the right ear, HIV-positive participants were affected with hearing change to a greater degree compared to HIV-negative participants at similar cisplatin doses. At 8000 Hz HIV-positive participants were likely to undergo an 11-unit increase (*β-coefficient* 10.89) in hearing threshold at 200 mg/m^2^ cisplatin dose (p ≤ 0.05), compared to a 4-unit increase (*β-coefficient* 4.29) among the HIV-negative participants exposed to the same dose (S5, S6 Files). It was also observed that from 8000Hz to 14000Hz, the effect of cisplatin dose on hearing was significant as the dose increased, although there wasn’t a consistent increase in the magnitude of change. Similar observations were made for the left ear (S7, S8 Files). A clustering of the extended-high frequencies (9000Hz– 14 000Hz) is evident among the HIV-positive strata (Fig 6, left and right ear) with the maximum change in hearing threshold observed at the 200 mg/m^2^ dose. The Tobit regression suggests that a combined model with age and HIV may better reflect the realities of the real-world scenario in measuring ototoxic shift.

Presenting with other co-morbidities such as diabetes and hypertension, did not significantly increase a participant’s risk of presenting with hearing loss during and after the course of cisplatin treatment (p = 0.71 in the left ear and p = 0.39 in the right ear), despite there being a steady increase in the number of these participants presenting with hearing loss at each successive audiological evaluation.

## Discussion

This study highlighted the adverse audiological impact of cisplatin in the course of cervical cancer treatment, with HIV-positive participants exhibiting a greater severity in hearing loss than their HIV-negative counterparts. By the end of the six-month follow up period, all of the participants (100%) presented with an ASHA [22] significant ototoxic change whilst 84% presented with a significant ototoxic change when the NCI-CTCAE v5 grading scale was used. The ASHA criteria, being binary and conservative, is considered an excellent starting point but has limited clinical utility in quantifying the degree of toxicity [33]. Therefore, the Health Professions Council of South Africa (HPCSA) [34] recommends the sequential use of ASHA [22] criteria and then the NCI-CTCAE grading scale [30] to grade the severity of the ototoxic change which relates to the functional impact on the patient’s quality of life.

The percentage of significant ototoxic change in the current study, being higher than previously reported in South Africa [35], and other international studies [36–42], may be attributed to the use of extended high-frequency audiometry in the current study. However, there is still a higher percentage of ototoxic change in the current study compared to other studies that also utilized extended high-frequency audiometry [9, 40], which could be due to differences in the study population, such as the high incidence of HIV and the use of ARTs.

HIV and the subsequent use of ARTs aggravate cisplatin-ototoxicity, with there being a significant association by the three and six-month post treatment evaluations. The synergistic effect of ARTs and cisplatin on the auditory system may also account for a lower cumulative cisplatin dosage resulting in a more rapid range in the hearing thresholds in the HIV-positive group than the HIV-negative group. A synergistic effect on hearing, implies that the resultant hearing loss is greater than the sum of the hearing losses that occur from any one co-morbidity or ototoxic medication alone [43]. The ototoxic effects of cisplatin on the auditory system could have been worsened by the use of certain ARTs, which through mitochondrial toxicity, may result in accelerated changes on the cochlear and/or central auditory system [21], or through the proposed direct effects of the virus on the cochlea and auditory nerve [44]. However, since all HIV-positive participants were on ARTs, it is impossible to disentangle the effect of the disease process from that of the ototoxic effect of ARTs.

van der Westhuizen et al. [44] reported that HIV may result in auditory dysfunction through the following mechanisms: “direct effects of the virus on the central nervous system and 8^th^ cranial nerve, opportunistic infections associated with hearing loss and ototoxicity as a result of highly active antiretroviral treatment (HAART), and medication administered in the treatment of opportunistic infections” (p. 37). Whilst conductive hearing loss and outer/middle ear infections are common in HIV-infected individuals, the low incidence of these otologic conditions in the current study (as reflected by the normal otoscopic and tympanometry results) highlights the benefits of the use of ARTs in preventing upper respiratory tract infections, which predispose affected individuals to middle ear infections and consequent conductive hearing loss. Additionally, it may indicate a lower viral load in this cohort due to the use of ARTs for a minimum of one year, which has proven to improve the impaired functioning of the immune system. However, it was not possible to identify the participants’ viral load, CD4 count, and treatment adherence in the current study, as this information was not captured in the hospital medical file due to participants receiving their ART from other decentralized local clinics. Additionally, participants were not knowledgeable of this information. As neurological testing was not conducted in the current study, no comment can be made about the direct action of HIV on the auditory nerve contributing to hearing loss.

Our findings on cumulative cisplatin dosage effects concur with literature [9, 42, 45, 46] which reports higher cumulative doses to have a greater impact on hearing thresholds, with more patients in the high-dose group than the low-dose group reflecting a Grade 2/3 category of the NCI-CTCAE grading scale at the six-month post treatment follow-up. While Whitehorn et al. [35] reported that median cumulative dosages of 180.70 mg/m^2^ and 236.84 mg/m^2^ were likely to result in ototoxic hearing loss for the ototoxicity-free and ototoxicity groups, respectively, our study revealed that increasing cumulative dosages from 150mg/m^2^ have increasing effects in the high and extended high-frequency region bilaterally, with a plateau effect seen in the left ear at 250mg/m^2^, with no further change in hearing thresholds at increased dosages.

Our findings on progressively worsening pure-tone hearing thresholds, evident in the extended frequency range, supports previous literature [9, 14, 40, 47]. However, it was shown that there is a tendency for the standard deviation of pure tone measurements in participants with otologically normal ears to increase with increasing frequency (Hz) and increasing age [48]. Furthermore, the number of ‘no responses’ increased with increasing frequency at each subsequent evaluation, resulting in ‘less’ ears for analysis in this frequency range (12500Hz- 20000Hz). An extrapolation of missing data was considered unreliable, as these frequencies (i.e. 16, 18, and 20kHz) were omitted from the regression analysis. However, the steadily increasing number of “no responses” in the extended high-frequency range is clear evidence of the deterioration of hearing thresholds in this frequency range.

Deterioration of hearing thresholds in the extended high-frequency range does not generally negatively affect word recognition scores, as is evident in the current study with more than 90% of the participants presenting with excellent word recognition scores within normal conversational levels (60 dBHL) at all audiological evaluations. These findings concur with Theunissen et al. [49], who reported that reduced hearing sensitivity in the extended high-frequency range may affect a participant’s well-being differently, as compared to hearing loss in the speech frequencies. A hearing loss up to and including 4000Hz may cause reduced speech intelligibility in noisy environments, while a hearing loss in the higher frequencies (>4000Hz) may negatively affect some speech sounds such as ‘f’, ‘s’ and ‘th” as well as sounds perceived in nature and music. However, it is of crucial importance for a person to be able to detect sounds between 5000Hz and 9000Hz because these sounds allow for adequate acoustic perception, as they “guarantee a good part of speech intelligibility by favoring consonant discrimination and speech recognition” (p. 39) [50]. This is of paramount concern as participants in the current study may, therefore, have "hearing for speech" but experience difficulty with specialized listening i.e. music, environmental sounds, and word recognition, in challenging communication settings. Therefore, complaints of reduced hearing sensitivity were lower than diagnosed hearing loss at all audiological evaluations, as these participants were able to hear speech for communication.

These results also, therefore, concur with the large number of participants presenting with ‘No Change’ when the NCI-CTCAE grading scale is used at the mid-cycle audiological evaluation, as this scale reflects the functional impact the ototoxic change has on the patient’s communication [51]. As time progressed, the number of participants reflecting ‘No Change’ decreases and concurs with the increasing complaints of reduced hearing sensitivity. As mild hearing loss or even a mild-to-moderate hearing loss was most common in the right and left ears respectively, participants may not perceive hearing difficulties due to them also subconsciously using visual cues to supplement their hearing. Furthermore, hearing loss is usually suspected when the individual experiences symptoms or if detected only during a hearing screening or when a communication problem becomes evident [52]. Therefore, a more accurate description of the functional impact of ototoxic hearing loss could possibly be obtained through the use of speech-in-noise tests, as reflected in the findings of Blankenship et al. [53]. Herein, a significant correlation between EHF hearing and speech-perception-in-noise was reported. Furthermore, patient self-report of communication difficulties was indicated not to be predictive of hearing loss or speech-in-noise performance.

Complaints of tinnitus increased during treatment and thereafter steadily decreased from the one-month post-treatment assessment; thus, indicating a resolution of tinnitus post-treatment. The resolution of tinnitus is in keeping with Bokemeyer, Berger [42], Melamed, Selim [54], Reddel, Kefford [55], who reported that tinnitus resolved or decreased in some participants receiving chemotherapy after a median duration of 6 months (range 1–18 months). As more than 70% of the participants described the tinnitus as high-pitched, the current authors postulate that this decrease in the number of participants reporting tinnitus maybe due to the participants no longer being able to hear these high frequencies (possibly in the extended high-frequency range) even at extremely high-intensity levels. This postulation would be consistent with our findings of an increasing number of ‘No responses’ in this frequency range. However, as no tinnitus matching tests were conducted during the assessment, this is purely conjecture and is possibly an area requiring further investigation. Another postulation would be that an increase in the stress and anxiety associated with receiving cisplatin chemotherapy, as well as thoughts about their prognosis, may have resulted in tinnitus, as the number of participants reporting tinnitus increased during treatment but decreased post-treatment. This would, therefore, be in keeping with the findings of Hasson, Theorell [56], who reported a linear association between tinnitus and the magnitude and duration of stress. Melamed et al. [54] and Reddel et al. [55] also indicated that while the reversibility of tinnitus was common, threshold abnormalities persisted.

The reported progression of hearing loss for six months after cessation of cisplatin chemotherapy in this study is an indication of cisplatin retention in the cochlear indefinitely [57] and, therefore, exemplifies the need for continued ototoxicity monitoring for patients who have received cisplatin chemotherapy considering the permanency of a sensorineural hearing loss. Sensorineural hearing loss, predominantly in the high and extended high-frequency range, is a common characteristic of cisplatin related-ototoxicity due to the effect of cisplatin on the outer hair cells in the basal turn of the cochlear, resulting in an initial elevation of high-frequency audiometric thresholds, followed by a progressive loss in the lower frequencies with continued therapy [58, 59]. This type and pattern of hearing loss correlates clinically with the described histopathological changes ascribed to cisplatin ototoxicity, which shows damage to the outer hair cells, supporting cells, marginal cells of the stria vascularis, spiral ligament and the spiral ganglion cells [60].

Furthermore, the increasing number of abnormal DPOAE findings in each ear with subsequent audiological evaluations is congruent with sensorineural hearing loss. Surprisingly, despite there being a significant decrease in the DPOAE amplitudes at all frequencies bilaterally, the number of abnormal DPOAE results are lower than would be expected when considering the increasing number of participants presenting with ototoxic hearing loss. These findings concur with Teotia et al. [61], who also reported a lower number of abnormal DPOAEs. The authors explained this finding, that while significant ototoxic change criteria for HFA are well established with excellent specificity and sensitivity, the criteria used for DPOAE interpretation is based on studies of DPOAE variability in healthy young adults. The current authors also postulate that the normal DPOAE results may be due to most participants in the study presenting with normal hearing or mild-to-moderate degrees of hearing loss in the conventional frequency range, and DPOAES are generally absent in frequency regions with pure tone thresholds greater than 50 dB [59]. Furthermore, the DPOAE measurement was limited to the conventional frequency range, which is the frequency range for “hearing for communication”.

Factors that have been overlooked in previous studies investigating the effects of cisplatin, namely co-morbidities such as diabetes, hypertension, HIV infection, and the subsequent exposure to other ototoxic medication, have been considered in the current study as many women in South Africa present with such comorbidities [62]. Due to the median age of the study sample being 52 years and the known impact of aging on the auditory system, age was treated as a confounding factor. Presenting with non-communicable diseases such as diabetes and hypertension did not significantly influence cisplatin ototoxicity, despite these conditions affecting similar auditory pathway structures and resulting in a sensorineural hearing loss. However, these results could be attributed to the low number of participants presenting with diabetes and hypertension and therefore statistical significance was not achieved in our study.

The strength of this study is evident by the selection of the study design, which allowed for the prospective observation of cisplatin’s effect on hearing in individuals within the cohort over time [63]. The use of prescribed guidelines for ototoxicity monitoring and internationally accepted normative data for analysing the audiological results further enhanced the scientific rigour of this study. An additional strong point of this study is the extremely low attrition rate in relation to the sample size. However, being a single institution study conducted at a public health facility, the findings of the current study cannot be generalised to the larger population of women with cervical cancer. Additionally, following patients for a longer duration may have yielded a more significant effect of the drug, considering the retention of cisplatin in the cochlear indefinitely [57]. The study did not investigate the impact of ototoxicity on quality of life of affected participants.

## Conclusion

This prospective cohort study, being the first in South Africa and the African continent, has revealed cisplatin’s detrimental effect on hearing with an even lower cumulative dosage of the treatment, possibly due to the large prevalence of HIV and subsequent use of ARTs. To prevent or avoid further deterioration in the quality of life of women with cervical cancer at a time when they are probably living in both physical and mental distress, early and effective identification of ototoxicity is crucial. Audiological monitoring should, therefore, be an essential part of the holistic management of patients receiving cisplatin chemotherapy, irrespective of cancer type, particularly in South Africa and other countries, which have a high burden of HIV. For patients who experience asymptomatic hearing loss but require continuation of cisplatin treatment, intensive audiological monitoring needs to be implemented with possible considerations of utilising oto-protective medication intra-tympanically. The findings of this current study will increase healthcare personnel’s awareness of the ototoxic effect of cisplatin and the need for comprehensive audiological monitoring with the potential of improving the quality of life for oncology patients. The potential for further investigation into chemotherapeutic regimens with minimal or no ototoxic effects is also warranted.

## Supporting information

S1 File Criteria for determining the presence of a significant ototoxic shift (ASHA) and grading of severity (NCI-CTCAE). (TIF)

S2 File Number of no responses at the various frequencies at each audiological evaluation. (TIF)

S3 File Association between pure tone frequency threshold measurements in the right ear and cumulative dosage of cisplatin, adjusted for the effects of age and HIV seropositivity. (TIF)

S4 File Association between pure tone frequency threshold measurements in the left ear and cumulative dosage of cisplatin, adjusted for the effects of age and HIV seropositivity. (TIF)

S5 File Association between pure tone frequency threshold measurements in the right ear and cumulative cisplatin dosage in the HIV-negative cervical cancer cohort (adjusted for the effects of age). (TIF)

S6 File Association between pure tone frequency threshold measurements in the right ear and cumulative cisplatin dosage in the HIV-positive cervical cancer cohort (adjusted for the effects of age). (TIF)

S7 File Association between pure tone frequency threshold measurements in the left ear and cumulative cisplatin dosage in the HIV-negative cervical cancer cohort (adjusted for the effects of age). (TIF)

S8 File Association between pure tone frequency threshold measurements in the left ear and cumulative cisplatin dosage in the HIV-positive cervical cancer cohort (adjusted for the effects of age). (TIF)
