# Supplementary figures and images for: Cisplatin-associated ototoxicity amongst cervical cancer patients: A prospective cohort study in south Africa
Source: PLoS One. 2023 Apr 4;18(4):e0283639. doi: 10.1371/journal.pone.0283639 (PMC10072443; doi:10.1371/journal.pone.0283639)

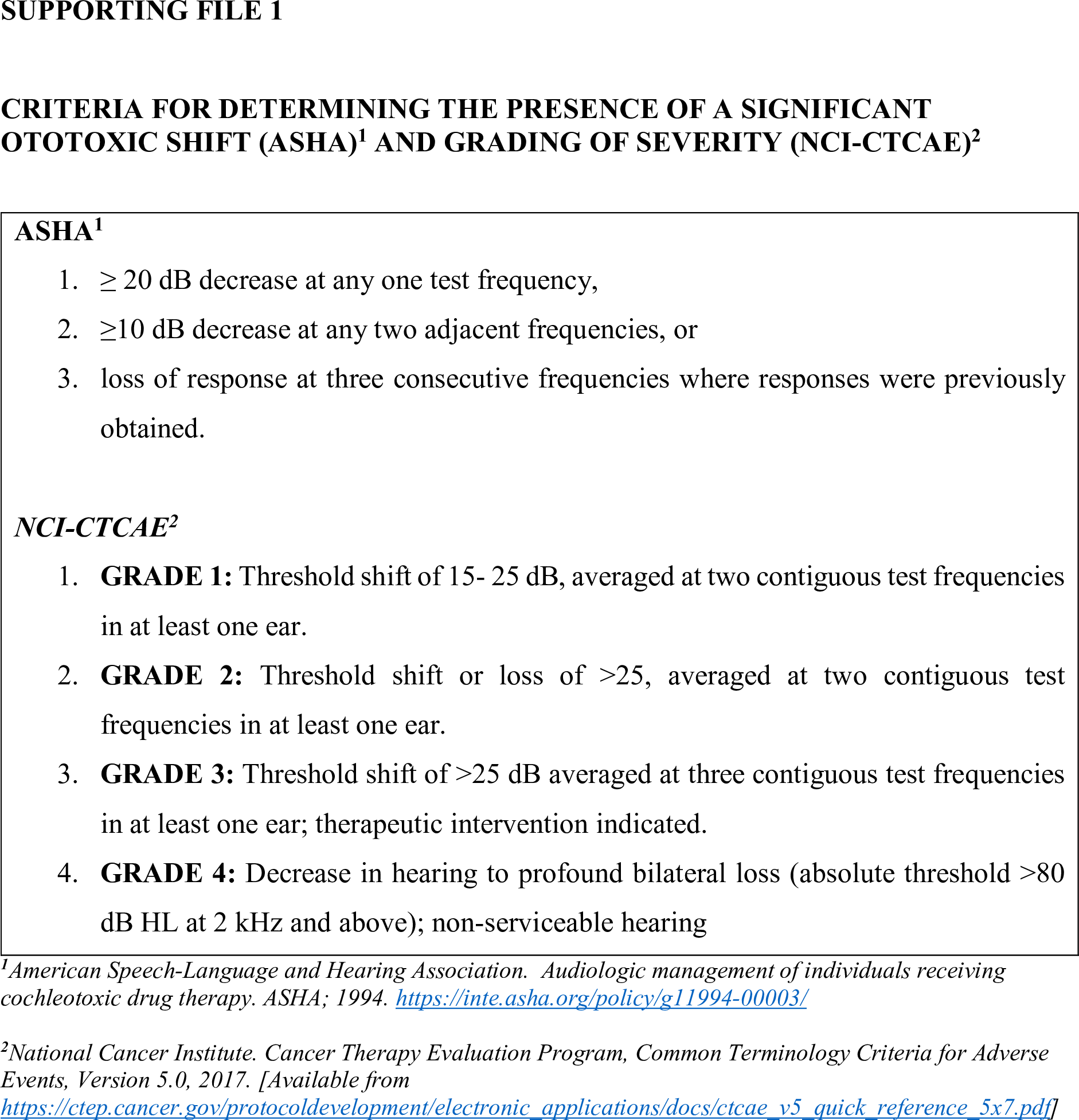

Supplement: S1 File — (TIF) [file pone.0283639.s001.tif]

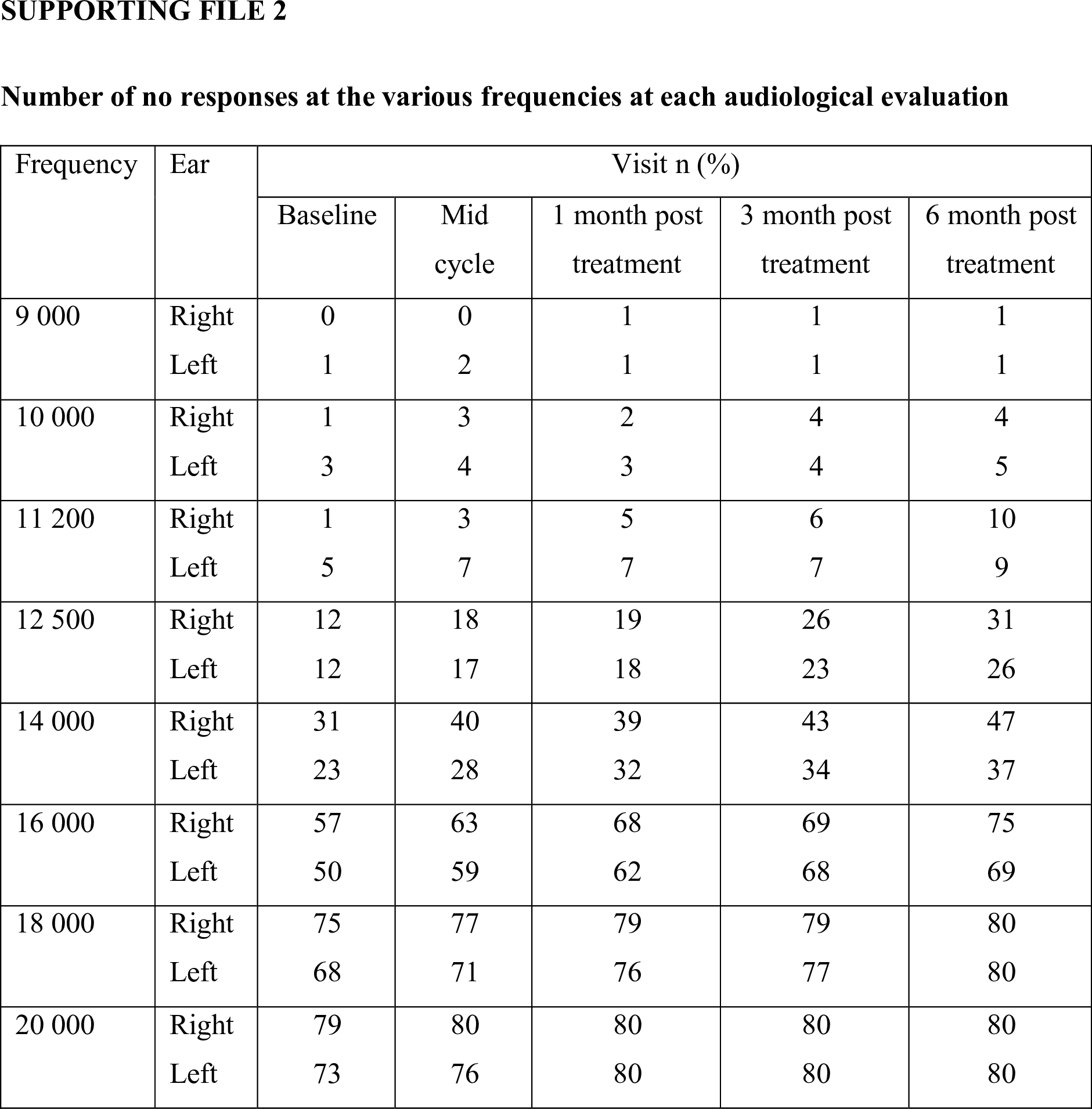

Supplement: S2 File — (TIF) [file pone.0283639.s002.tif]

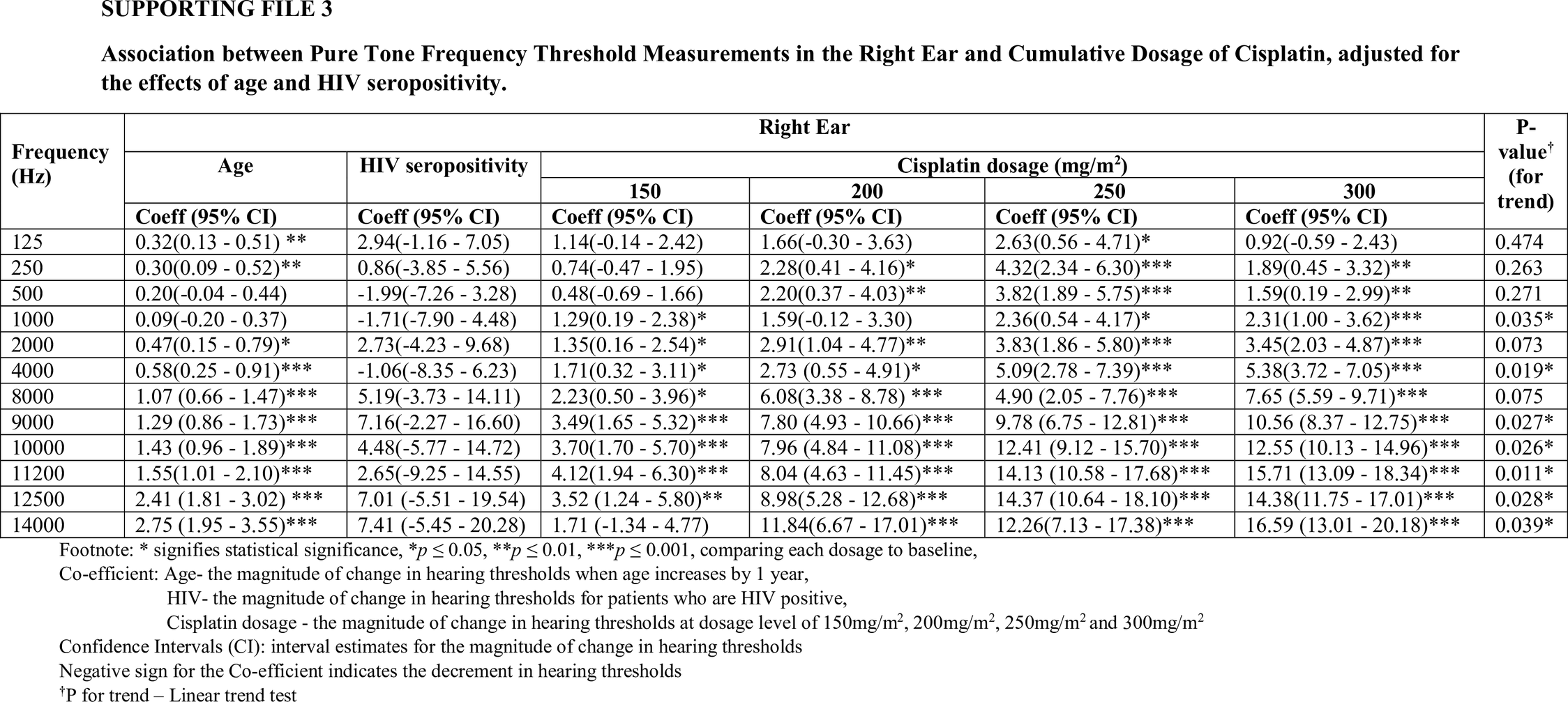

Supplement: S3 File — (TIF) [file pone.0283639.s003.tif]

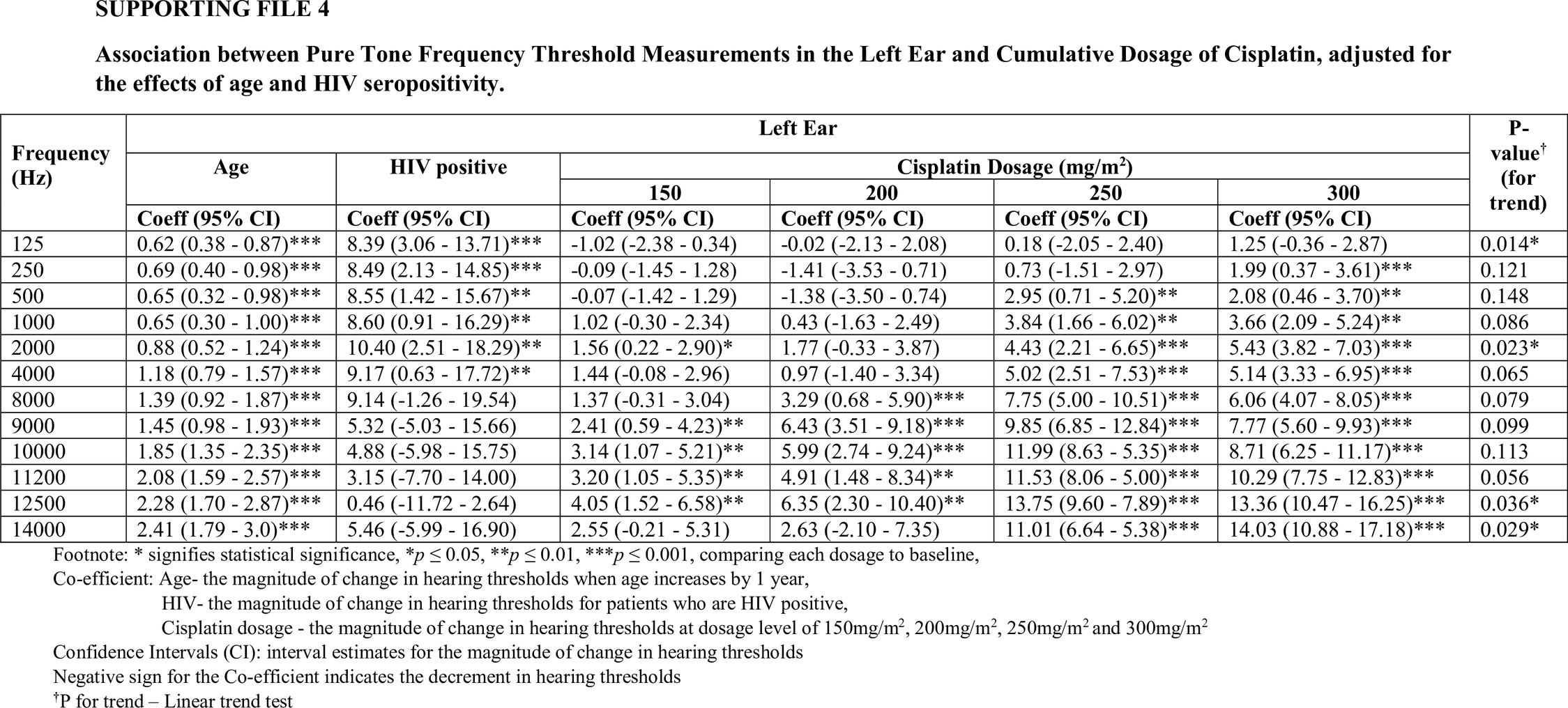

Supplement: S4 File — (TIF) [file pone.0283639.s004.tif]

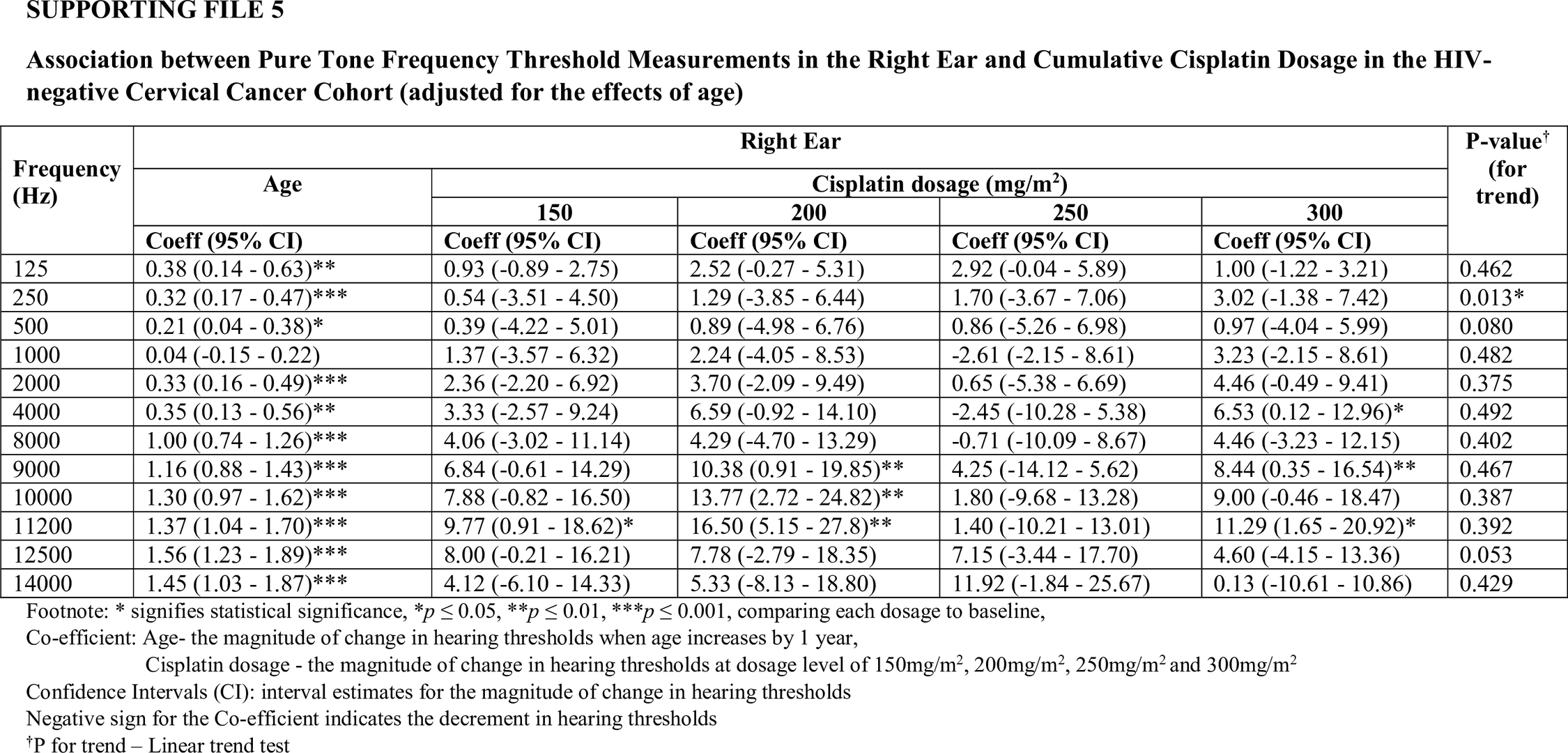

Supplement: S5 File — (TIF) [file pone.0283639.s005.tif]

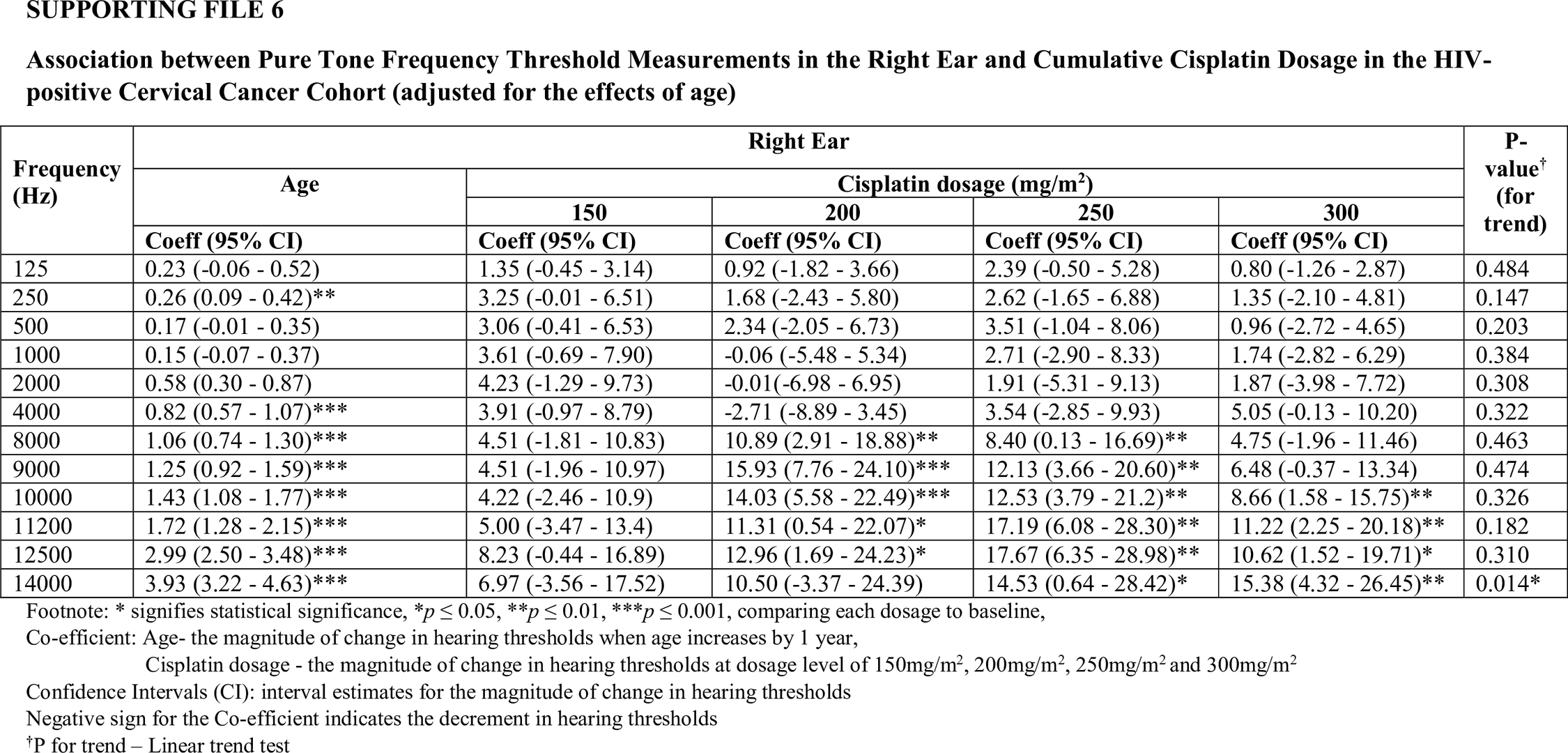

Supplement: S6 File — (TIF) [file pone.0283639.s006.tif]

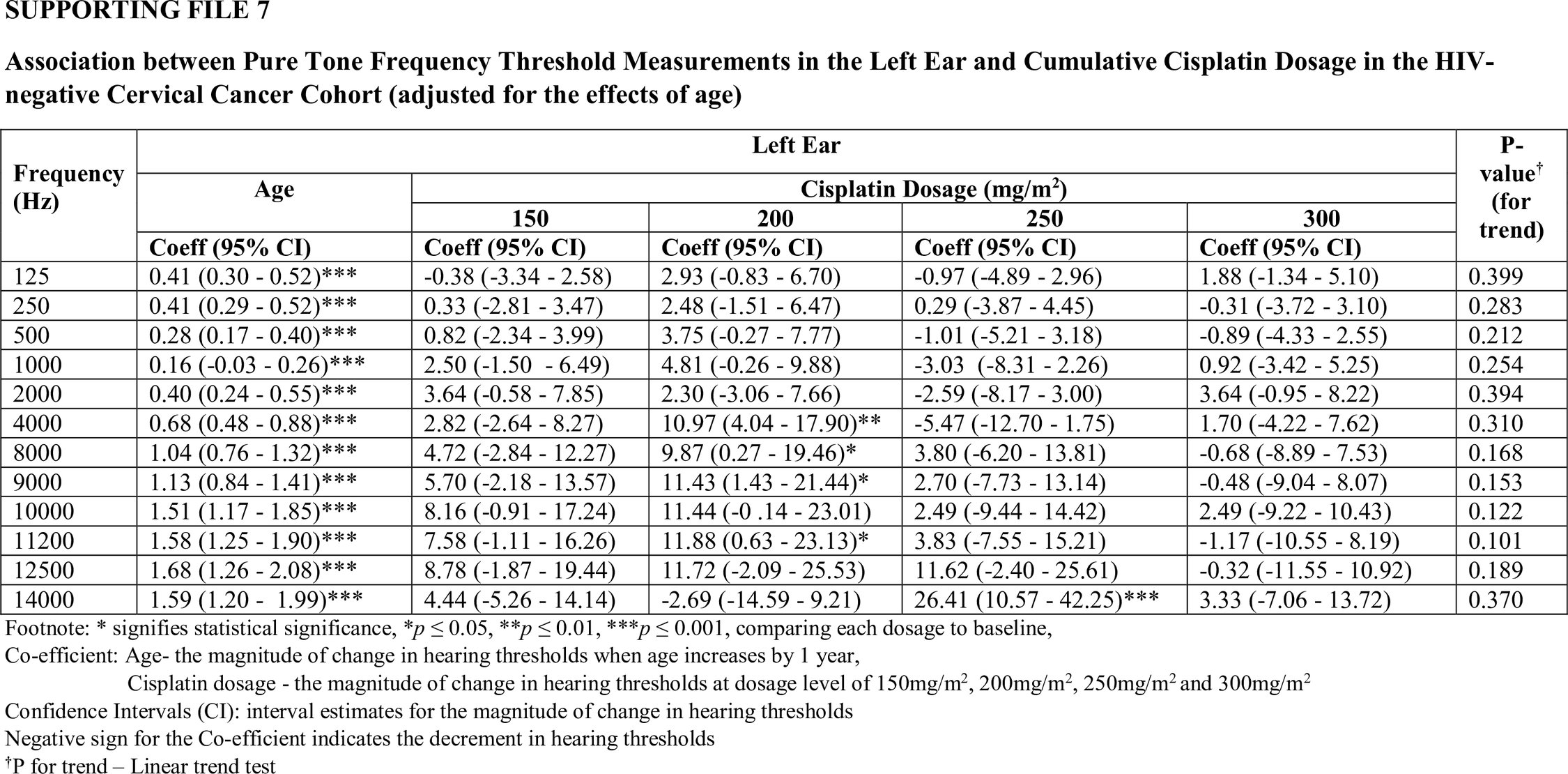

Supplement: S7 File — (TIF) [file pone.0283639.s007.tif]

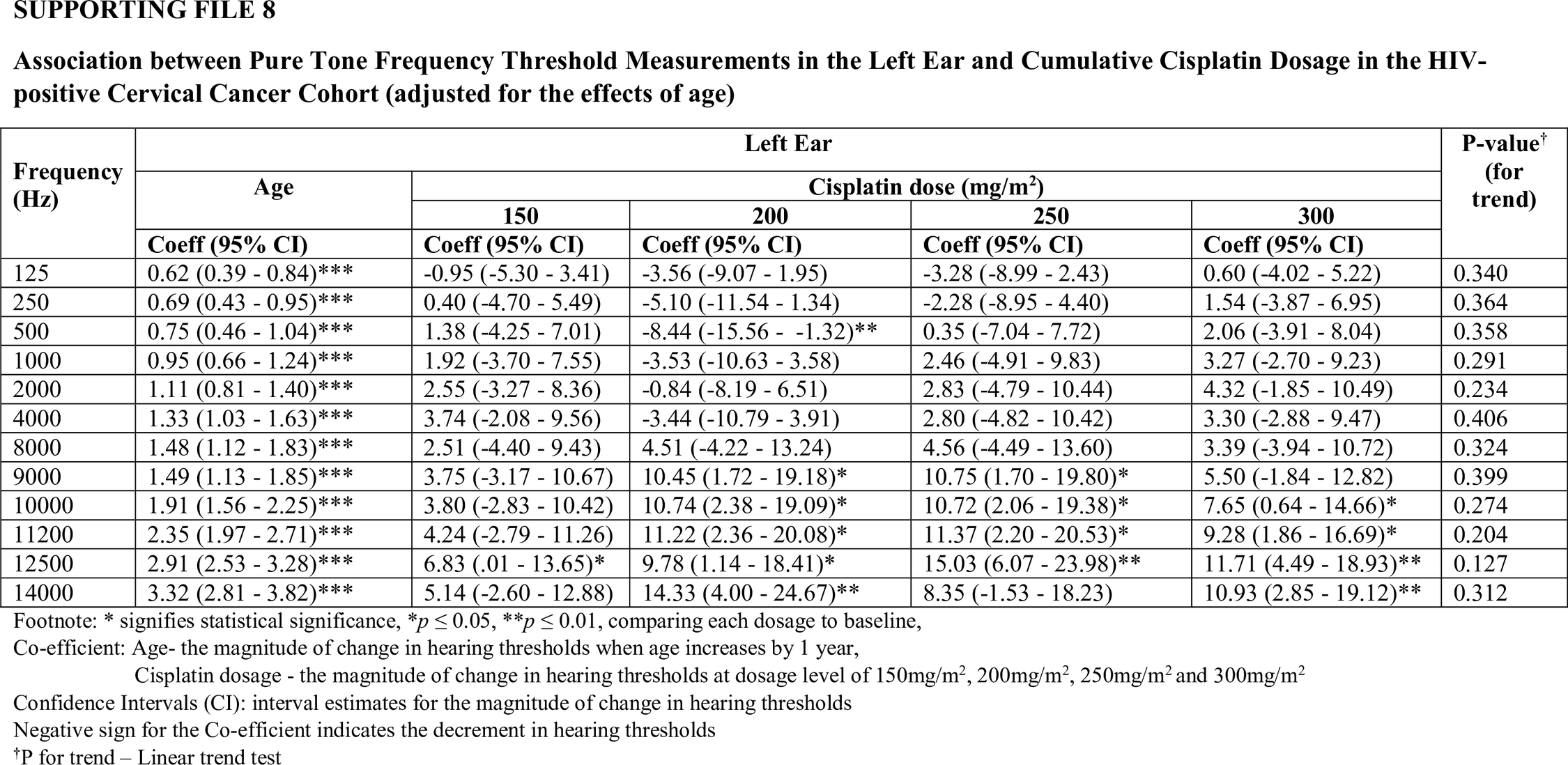

Supplement: S8 File — (TIF) [file pone.0283639.s008.tif]
